# Acupuncture for Chronic Pain Management in Haemophilic Arthropathy: A Systematic Review

**DOI:** 10.1111/hae.70109

**Published:** 2025-08-21

**Authors:** Andrea Demeco, Marco Gusai, Maria Francesca Masi, Antonio Frizziero, Cosimo Costantino

**Affiliations:** ^1^ Physical and Rehabilitative Medicine Department of Medical and Surgical Sciences University of Catanzaro “Magna Graecia” Catanzaro Italy; ^2^ Department of Medicine and Surgery University of Parma Parma Italy; ^3^ Surgical and Dental Sciences Department of Biomedical University of Milan Milan Italy

**Keywords:** acupuncture, pain, rehabilitation

## Abstract

**Introduction:**

Haemophilia is a severe hereditary bleeding disorder affecting ∼1 in 5000 males, caused by deficiencies in coagulation factors VIII or IX. Chronic pain from haemophilic arthropathy (HA), especially in target joints, reduces quality of life (HRQOL), range of motion (ROM) and daily function. While conventional treatment includes replacement therapy and rehabilitation, acupuncture has emerged as a complementary approach with potential pain‐relief benefits and fewer side effects.

**Aim:**

To evaluate the effectiveness of acupuncture in reducing chronic pain and improving function and quality of life in people with haemophilia (PWH).

**Materials and Methods:**

This systematic review followed PRISMA guidelines (PROSPERO: CRD42024567714). Databases searched: PubMed, Scopus, Web of Science. PICO framework: (P) haemophilic patients, (I) acupuncture, (O) pain management. Studies on other coagulation disorders or non‐acupuncture therapies were excluded. Methodological quality was assessed using the JBI score.

**Results:**

Of 514 records (464 PubMed, 40 Scopus, 10 Web of Science), 26 duplicates were removed, and 350 titles were screened. Seven met the inclusion criteria; after quality assessment and availability check, four studies were included. A total of 37 patients (mean age 41.4 years) reported meaningful pain reduction (VAS), reduced analgesic use and improved HRQOL. No significant bleeding events were reported.

**Conclusion:**

Acupuncture may provide effective pain relief for haemophilic arthropathy with minimal side effects. However, larger, high‐quality studies are needed to confirm its clinical benefits.

## 1 Introduction

Haemophilia is the most common severe hereditary haemorrhagic disorder; its prevalence is estimated to be 1 in 5000 males, the incidence 1 in 1333 live male births [1, 2]. Haemophilia A and B result from clotting factor (CF) VIII and IX deficiency/dysfunction, respectively [2].

Hence, people with haemophilia (PWH) cannot produce sufficient thrombin and become dependent on the tissue factor (TF) pathway, due to excessive bleeding tendency after minor trauma or even spontaneously. Bleeding severity corresponds to the percentage of active circulating CF: ≤1% is classified as severe haemophilia, 2%–5% as moderate, 6%–40% as mild, but individual profile plays a crucial role in personalised treatment [3].

The most significant preventable cause of morbidity in PWH is degenerative joint disease due to recurrent haemarthrosis typically in the ankle, elbow and knee (target joints), susceptible to progressive arthropathy [4].

Chronic pain (CP) from haemophilic arthropathy (HA) is a widespread issue in PWH, negatively affecting their function, influencing health‐related quality of life (HRQOL) [5].

Treatment involves replacing deficient CF, on demand or prophylactically.

Due to the complexity of musculoskeletal alterations, treatment must be comprehensive to meet physical and biopsychosocial demands individually. Physiotherapy and TENS are common approaches for managing CP and HA. However, because of persistence of joint discomfort and pain, many PWH seek alternative therapies, such as acupuncture, as a viable treatment option [6].

Since the National Institutes of Health determined acupuncture effective for postoperative dental pain, interest in acupuncture has grown significantly as an integrative or complementary pain therapy [7]. Traditionally believed to restore normal flow of energy (qi) in the body, acupuncture has shown in modern research to impact central and peripheral nervous systems, releasing endogenous opioids, serotonin and norepinephrine, affecting nociceptors, inflammatory cytokines and other physiological mechanisms, altering pain perception [8, 9, 10, 11]. Despite the mechanism of acupuncture being unclear, recommendations are cautious, and most evidence is of low to moderate quality [12].

However, acupuncture has been suggested as an effective therapeutic strategy for pain relief in HA, associated with fewer adverse effects compared to conventional approaches [13]. It has been proven effective in a number of studies to control pain, and improve movement of joints by releasing the shortening of involved muscles [14, 15, 16].

Therefore, the aim of this study is to evaluate the effectiveness of acupuncture in reducing CP, improving joint function, quality of life (QOL) and satisfaction in PWH.

## 2 Materials and Methods

### 2.1 Search Strategy

This systematic review was made according to PRISMA‐NMA and Cochrane Handbook for Systematic Reviews of Interventions guidelines and was registered in PROSPERO: CRD42024567714. Two authors independently searched the English bibliography across databases: PubMed, Web of Science and Scopus. Boolean operators ‘AND’/‘OR’ combined keywords encompassing ‘haemophilia, hemophilia, bleeding disorder, factor VIII deficiency, factor IX deficiency, acupuncture, needling, dry needling’, as shown in Table 1.

**TABLE 1:** Search strategy.

**PUBMED**:
(((hemophilia) OR (haemophilia) OR (bleeding disorder) OR (factor VIII deficiency) OR (factor IX deficiency)) AND ((acupuncture) OR (needling) OR (dry needling)))
**SCOPUS**:
(TITLE‐ABS ( ‘'hemophilia'’ ) OR TITLE‐ABS ( ‘'haemophilia'’ ) OR TITLE‐ABS (‘'bleeding disorder'’) OR TITLE‐ABS ( ‘'factor viii deficiency'’) OR TITLE‐ABS ( ‘'factor ix deficiency'’ )) AND (TITLE‐ABS ( ‘'acupuncture'’ ) OR TITLE‐ABS ( ‘'needling'’ ) OR TITLE‐ABS ( ‘'dry needling'’ ))
**WEB OF SCIENCE**:
(TI = ((hemophilia) OR (haemophilia) OR (bleeding disorder) OR (Factor VIII deficiency) OR (factor IX deficiency))) AND TI = ((acupuncture) OR (needling) OR (dry needling))

### 2.2 Selection of Articles

PICO criteria were: (P) People with hemophilia A or B; (I) acupuncture; (C) traditional techniques (physiotherapy, cryotherapy, non‐steroidal anti‐inflammatory drugs, extracorporeal shock wave therapy), TENS; (O) pain intensity, using VAS, QOL scale, using the Haem‐a‐Qol, joint health, using the Haemophilia Joint Health Score (HJHS), degree of satisfaction and expectations. Articles satisfying the following inclusion criteria were selected: PWH and HA treated with acupuncture; full‐text availability; English‐written; case‐control studies, RCTs, case reports. The exclusion criteria were: studies investigating alternative coagulation disorders or utilising no‐acupuncture treatments, non‐English language; (4) no full‐text.

### 2.3 Data Extraction

Two independent reviewers screened titles and abstracts to eligibility according to the selection criteria. Articles lacking clarity regarding inclusion criteria underwent full‐text review. Data extraction was performed utilising a standardised Microsoft Excel sheet.

We gathered the following data: authors, publication year, nationality, number of patients included, age of patients, intervention, comparison, outcome measure and time‐point assessments, main findings.

### 2.4 Outcome Measures

Visual Analogue scale (VAS) was employed to assess pain intensity, ranging from 0 (‘no pain’) to 10 (‘worst possible pain’) [6, 13, 17].

To assess acupuncture's impact on participants' HRQoL, the Short Form Health Survey 36 (SF‐36) was administered at baseline and post‐intervention, including eight domains: physical functioning, social functioning, physical problems, emotional problems, mental health, energy/fatigue, pain and general health perceptions [13]. The self‐administered Haem‐a‐Qol questionnaire was also used, encompassing 10 domains (e.g., physical health, emotional well‐being, self‐perception) scored from 0% (worst) to 100% (best) [6].

The Haemophilia Joint Health Score (HJHS) assesses the health of joints most susceptible to bleeding in haemophilia (knees, ankles, and elbows), scoring a range from 0 to 124, with lower scores indicating minimal or no musculoskeletal changes and higher scores reflecting severe changes in all evaluated joints [13].

### 2.5 Quality Assessment/Risk of Bias

We assessed methodological studies' quality using the JBI critical appraisal checklist, available online, selecting the version in accordance with the study design analysed [18]. We used a ternary scoring (1 = yes, 0 = no/unclear) for each item. Two authors independently assessed quality scores, resolving disagreements through consensus discussion with a third reviewer.

Studies were categorized as high (85%–100%), moderate (60%–84%) and low quality (<60%).

## 3 Results

### 3.1 Evidence Synthesis

**FIGURE 1:**
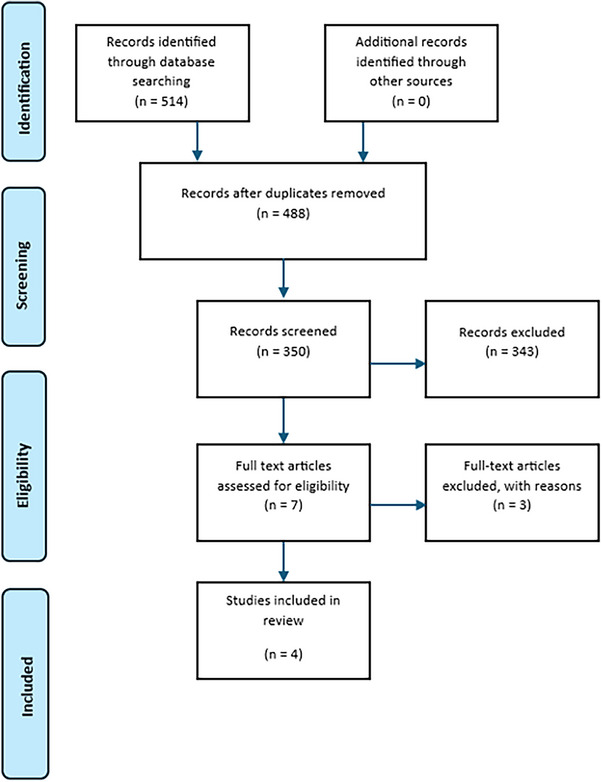
PRISMA flowchart.

Literature search generated 514 articles: 464 from PubMed, 40 from Scopus and 10 from Web of Science. Twenty‐six articles were excluded for being duplicates, and 350 were screened. Finally, seven fulfilled inclusion criteria; we excluded two after quality assessment and one for missing full‐text. Lastly, four studies were included in the systematic review (Figure 1).

A total of 37 patients (average of 41, 4 years of age) are included in the studies; the main findings are shown in Table 2.

**TABLE 2:** Main characteristics of the studies.

Articles	Study group	Control group	Intervention	Comparison	Outcome measure and time‐point assessments	Main findings
Lambing et al. [13]	N: 9 subject with severe, moderate or mild haemophilia A or B; mean age was 45 years, with a range of 28–63 years.	/	Subjects received acupuncture treatment twice weekly for 4 weeks, then weekly for 6 weeks, totaling 14 acupuncture treatment sessions.	/	Pain was recorded using the visual analogue scale (VAS) of 0–10. Subjects were asked to rate their average daily pain, including the highest level and lowest level achieved. Subjects recorded their use of pain medications, including the type of pain medication and daily frequency of usage. Quality of life (QOL) was evaluated using the SF‐36 tool, completed prior to the study, and repeated after the last acupuncture session.	Four out of the nine participants reported at least a 50% reduction in their pain scores on the visual analogue scale (VAS) following the acupuncture program. The collective quality of life (QOL) scores demonstrated an improvement in seven of the nine QOL domains.
Oliveira et al. [6]	N: 15; mean age 43,33; men with moderate or severe haemophilia A or B and between the age of 18 and 65 years, with chronic pain due to chronic arthropathy.	N: 13; mean age 32,77; men with moderate or severe haemophilia A or B	Subjects received weekly unilateral 20‐min sessions of acupuncture, with a total of 5 consecutive sessions.	Participants were treated with 5 consecutive weekly sessions of transcutaneous electrical nerve stimulation (TENS), each lasting 20 min.	Quality of life was evaluated using the self‐administered Haem‐a‐Qol instrument, a 10‐domain scale ranging from 0 to 100. The questionnaire was completed before the first and after the last session of each protocol. The Hemophilia Joint Health Score (HJHS) instrument, a 0–124 scale, was used to evaluate joint health in the ankles, knees and elbows at baseline and at the end of the treatment period. A visual analogue scale (VAS) was also applied for pain evaluation.	Acupuncture led to a remarkable 60.71% reduction in pain intensity, as measured by the Visual Analogue Scale (VAS) (initial mean VAS = 5.6 vs. final mean VAS = 2.2; *p* = 0.000). Both the acupuncture group and the control group experienced a statistically significant decrease in average VAS scores before and after treatment (*p* = 0.005). The HJHS did not show any significant improvement in either group. However, there was a significant difference (*p* = 0.004) between the two groups when it comes to their degree of satisfaction.
Rosted and Jørgensen [19]	A 38‐year‐old patient with severe haemophilia (baseline factor VIII<0.01 IU/mL) and severe arthropathy of both knees, elbows and ankles.	/	Five acupuncture treatments.	/	Evaluation of referred pain and medication use.	Significant reductions in pain intensity, frequency and medication intake were observed.
Wallny et al. [17]	N: 12 patients with a factor VIII activity <1% and at least one painful arthropathy in both lower extremities.	/	Cranial acupuncture administered weekly over 15 weeks.	/	By means of a visual analogue scale (VAS), they were asked to evaluate the pain in the large joints of the upper and lower extremities before and after the therapy. Furthermore, a clinical orthopaedic examination was performed, assessing range of motion, swelling and warmth of the target joints.	Ten of 12 patients showed an improvement in their pain perception. VAS could be reduced from 6.8 to 5.0. Six patients were able to reduce their pain medication intake.

### 3.2 Intervention Protocol and Technical Features

Acupuncture's intervention methods showed variations in terms of frequency, duration and approach to treatment, tailored to specific patient groups and objectives.

Lambing et al. patients received twice‐weekly sessions for 4 weeks, then once‐weekly for 6 weeks, totalling 14 sessions lasting 30–35 min. Some of acupoints were: SP6, LI11, Liv 3, LI4, GB34, ST36, Shen Men and Du 20. No electrical devices were utilised [13].

Oliveira et al. adopted a once‐weekly treatment approach (20 min) over 5 consecutive weeks. Needles were manually rotated until Deqi sensation, with insertion depths tailored to the participant's age and physical constitution. Some targeted acupoints were: ST40, ST41, ST32, GB41, LI4, LU9, LR3, SP6, LI11 and LR8 [6].

Rosted and Jørgensen treated elbow pain by inserting needles into tender points LU5 and LI11, rotated for 5 s, left in place for 5 min, and then removed. This method resulted in reduced pain and lower Oramorph intake. The patient received three similar treatments and managed well for 6 months before experiencing a slight flare‐up, controlled with two additional treatments. The additional point was LI10. Over 10 months, no relapses occurred. For knee pain, another session used acupoints SP9, SP10 and LR8. The same technique and needle type were applied, yielding significant pain reduction and decreased medication use, sustained by maintenance treatments continued approximately once every 3 months over 14 months [19].

Wallny et al. involved a different approach, focusing on cranial acupuncture administered once‐weekly over 15 weeks. Needles were placed at Du 18 in the galea, after which patients were instructed to move their lower extremities for at least 30 min [17].

In conclusion, each study showcases a diverse array of acupuncture techniques and acupoints (Tables SIII–SVI), each tailored to address specific patient conditions and treatment goals [6, 13, 17, 19].

### 3.3 Side Effects

Even in bleeders, incidence of acupuncture side‐effects is very low. No significant bleeding was observed, which is not surprising accounting the thin, stainless, disposable needle, as well as the region of insertion.

### 3.4 Pain Management

Lambing et al.’s participants reported average daily pain score along with their highest/lowest pain levels experienced during the day before and after treatment. Additionally, participants documented use of painkillers type and frequency. Four out of nine participants reported at least a 50% VAS score reduction post‐acupuncture, whereas seven out of nine patients demonstrated some degree of improvement. Results indicate a statistically significant improvement in seven out of the nine aspects of QOL [13].

Oliveira et al. reported 60.71% VAS (initial mean 5.6 vs. final mean 2.2; *p* = 0.000). Both the acupuncture and control groups reported a statistically significant decrease in VAS scores before and after treatment (*p* = 0.005). Although either group showed any statistically significant difference in HJHS (*p* = 0.005), so did they in satisfaction degree (*p* = 0.004) [6].

Rosted and Jørgensen reported a significant improvement in both pain intensity and frequency after treatment [19].

Wallny et al. reported that 10 out of 12 patients showed a significant improvement in their pain perception: VAS significantly decreased from 6.8 to 5.0 and six patients were able to reduce painkiller intake [17].

However, amongst the studies, acupuncture benefits appeared limited to the specifically treated joints (knee, hip and ankle), while there is no evidence of improvement in ROM or inflammation.

### 3.5 Study Quality

We opted for JBI critical appraisal checklist for Case for Case Report (one study) [19], JBI Case Series (two studies) [13, 17] and JBI for RCT (one study) [6].

One of the four studies considered were of high quality, the remaining three were of moderate quality, as shown in Table 3.

**TABLE 3:** Joanna Briggs Institute (JBI) critical appraisal checklist for RCT (one study), case report (one study) and JBI case series (two studies).

Article	Item 1	Item 2	Item 3	Item 4	Item 5	Item 6	Item 7	Item 8	Item 9	Item 10	Item 11	Item 12	Item 13	JBI score (%)
Oliveira et al. [6]	1	1	1	0	0	0	1	0	1	1	1	1	1	69.2%
Article	Item 1	Item 2	Item 3	Item 4	Item 5	Item 6	Item 7	Item 8	JBI score (%)					
Rosted and Jørgensen [19]	1	1	1	1	1	1	1	1	100%					
Article	Item 1	Item 2	Item 3	Item 4	Item 5	Item 6	Item 7	Item 8	Item 9	Item 10	JBI score (%)			
Lambing et al. [13]	1	1	1	0	0	1	1	1	1	1	80%			
Wallny et al. [17]	1	1	1	0	0	1	1	1	0	1	70%			

## 4 Discussion

The systematic review aimed to assess the efficacy of acupuncture in managing CP associated with HA. We examined the efficacy of various acupuncture protocols in alleviating CP, enhancing QOL, joint function and treatment satisfaction among PWH.

The studies included in this review suggest that acupuncture may provide pain relief and functional benefits in individuals with haemophilic arthropathy; however, findings should be interpreted with caution due to the small sample sizes and methodological limitations. Lambing noted an improvement in QOL, in addition to pain reduction, suggesting that acupuncture might have broader benefits beyond analgesia [13]. Moreover, Oliveira highlighted a marked pain intensity decrease, emphasizing acupuncture's positive impact on subjective pain perception [6]. Both Rosted and Jørgensen and Wallny et al. corroborated these findings, reporting a reduction in both pain intensity and frequency [17, 19].

The mechanisms underlying acupuncture's effectiveness in managing CP, while not yet fully understood, highlight its ability to modulate pain pathways through an extended combination of physiological effects. In the late 20th century, Bruce Pomeranz advanced the ‘neurohormonal theory’, proposing that acupuncture stimulates A‐delta and C afferent nerve fibres. This signalling propagates to key midbrain structures (Raphe Nucleus and Periaqueductal Grey) modulating pain suppression pathways, effectively inhibiting transmission within the spinal cord. Importantly, Pomeranz demonstrated that the analgesic effects of acupuncture could be blocked by naloxone, an opioid antagonist, providing direct evidence of involvement of endogenous opioid systems in acupuncture's mechanism of action [8].

Recent studies confirmed the central neurochemical changes involving the release of serotonin, beta‐endorphins and dynorphins, collectively reducing pain perception and suppressing inflammatory processes, aligning with Pomeranz's neural signalling theory and illustrating the broader biochemical effects acupuncture‐induced. Moreover, the increase in adenosine levels at needle insertion sites, as well as serotonin and norepinephrine production, further contributes to pain relief by local analgesia through A1 receptors [9] and by reducing inflammation [10].

At the spinal level, the gate control theory provides a compelling explanation for acupuncture's impact on nociceptive transmission, whereby the stimulation of A‐beta fibres inhibits pain signals by ‘closing the gate’ in the spinal cord [11]. Endogenous opioid release and neural pathway modulation, spanning spinal, mesencephalic and hypothalamic levels, add‐on the mechanical effect of acupuncture, which influences connective tissues through a process known as mechano‐transduction. Needle insertion and manipulation stimulate fibroblasts, remodel the extracellular matrix, and improve local blood circulation. This cascade not only reduces inflammation but also promotes tissue healing and alleviates pain [20, 21]. Furthermore, the improved circulation enhances nutrient and oxygen delivery to affected areas, while muscle relaxation alleviates stiffness, ultimately improving joint mobility and functional movement [22].

These results suggest acupuncture as a promising alternative for pain management in PWH, particularly due to the risks associated with nonsteroidal anti‐inflammatory drugs (NSAIDs) or other non‐invasive treatments, such as extracorporeal shock wave therapy (ESWT). PWH are especially vulnerable to haemorrhagic complications, and NSAIDs are known to exacerbate bleeding risks by inhibiting platelet function and damaging gastrointestinal mucosa [23]. ESWT is also contraindicated in coagulation disorders, because it can disrupt fragile blood vessels, resulting in internal bleeding [24, 25]. In contrast, acupuncture offers a non‐pharmacological approach that does not carry these risks, making it a safer option for pain control [26].

Acupuncture's safety profile in haemophilia is further supported by its adaptability to individual patient needs. Techniques can be modified to use superficial needling, avoid deep punctures and focus on non‐bleeding‐prone areas, minimising haemorrhage risk. Tailored approaches such as the use of smaller needles or acupressure in place of traditional needling are particularly beneficial, reducing tissue trauma and avoiding vascular injury [27, 28].

Other non‐pharmacological methods in HA, such as transcutaneous electrical nerve stimulation (TENS), also offer pain relief without increasing bleeding risk [29]. However, studies suggest that while TENS is effective, it is less successful than acupuncture [30]. TENS can indeed be substituted with electroacupuncture, a technique combining traditional acupuncture with electrical stimulation, enhancing the body's natural pain‐control mechanisms through more profound and sustained nerve stimulation [31]. In fact, research indicates that electroacupuncture may provide superior pain relief and longer‐lasting effects compared to TENS, stimulating both acupoints and deep tissues more effectively [30]. Cryotherapy is another commonly used method for reducing joint pain and inflammation by decreasing blood flow to affected areas, but it is mainly effective in acute settings and lacks long‐term benefits seen with acupuncture [32].

In addition, acupuncture has shown potential in complementing physiotherapy, particularly for managing HA [32]. Indeed, physical activity can benefit several chronic pain conditions, and acupuncture enhances it, making it an effective integrative treatment for joint health and functional mobility [33, 34, 35, 36, 37]. In fact, physical therapy alone can be hindered by pain during activity: combining it with acupuncture enhances overall treatment outcomes, allowing patients to engage more effectively in rehabilitation [35, 38].

The protocols described across the reviewed studies commonly involved weekly or biweekly sessions over a 4–10 week period, with some studies incorporating maintenance sessions every 2–3 months to support longer‐term effects [6, 13, 17, 19]. Treatment sessions typically lasted between 20 and 35 min, and manual needle stimulation was the most frequently used technique. These protocol characteristics reflect practice patterns observed in broader chronic pain literature [39] but their optimal configuration in the context of HA remains to be established through further research. Regarding acupoint selection, a consistent pattern emerged in which local points targeting affected joints (e.g., SP9, SP10, ST35, LR8 for the knee; LI11, LU5, LI10 for the elbow) were used alongside distal and systemic points such as LI4, Liv3, GB34, SP6 and Du20. These point combinations align with traditional acupuncture theory and have shown relevance in musculoskeletal pain models [40]. While no definitive conclusions can be drawn, these patterns provide a foundation for future protocol standardisation.

Future research should incorporate objective outcome measures such as musculoskeletal ultrasound (MSK‐US), magnetic resonance imaging (MRI) and biochemical markers of cartilage and bone metabolism to complement subjective assessments like the Visual Analogue Scale (VAS). While VAS provides valuable insight into patient‐perceived pain, it may not reflect underlying inflammatory activity or early joint degeneration. MSK‐US and MRI have proven effective in detecting subclinical synovitis, effusion and osteochondral changes in people with haemophilia (PWH), even when clinical symptoms are minimal or absent [4, 41].

Moreover, biochemical markers such as C‐terminal telopeptide of type II collagen (CTX‐II), cartilage oligomeric matrix protein (COMP) and bone‐specific alkaline phosphatase (BSAP) are being explored as promising tools to monitor joint health and disease progression in haemophilic arthropathy [42, 43]. Integrating these objective measures into clinical studies may provide a more comprehensive evaluation of treatment efficacy and disease status.

### 4.1 Limitations

Acupuncture studies face several limitations impacting their findings. A major issue is the small sample size that limits the generalisability of the results. Additionally, there is often lack of objective data collection. Furthermore, the subjective nature of outcomes can be influenced by factors beyond HA, like age differences between study groups.

Another important limitation lies in the susceptibility of acupuncture studies to placebo effects and the inherent difficulty in blinding both patients and practitioners. The subjective nature of outcomes such as pain perception—primarily assessed using the VAS—may be influenced by patient expectations and reporting bias [44]. While some trials attempt to implement sham acupuncture as a control, even minimal skin stimulation can activate mechanoreceptors and elicit neurophysiological responses, thus blurring the distinction between verum and placebo interventions [45, 46]. This makes it challenging to attribute therapeutic effects solely to acupuncture itself, rather than to contextual or psychosocial factors [47]. Moreover, the lack of double‐blinding in most included studies increases the risk of performance and detection bias, potentially inflating perceived treatment benefits [39].

## 5 Conclusion

In conclusion, this systematic review provides preliminary support for acupuncture as a potentially beneficial complementary therapy in the management of chronic pain associated with haemophilic arthropathy. Nonetheless, due to methodological limitations and small sample sizes, further high‐quality research is required to substantiate these findings. Through the modulation of central and peripheral pain pathways, acupuncture offers a non‐pharmacological alternative with minimal risk of adverse effects—particularly relevant for people with haemophilia who are at elevated risk from conventional analgesics. Integrating acupuncture with standard treatment protocols, including physiotherapy and joint monitoring, may enhance functional recovery and quality of life.

## Conflicts of Interest

The authors declare no conflicts of interest.

## Supporting information




**Supporting File**: hae70109‐sup‐0001‐SuppMat.docx

## Data Availability

Data is available from the corresponding author on reasonable request.
