# Supplementary material for: Acupuncture for Chronic Pain Management in Haemophilic Arthropathy: A Systematic Review
Source: Haemophilia. 2025 Aug 21;31(5):865–73. doi: 10.1111/hae.70109 (PMC12462551; doi:10.1111/hae.70109)
Supplement: Supplementary file 1 — Supporting File: hae70109‐sup‐0001‐SuppMat.docx [file HAE-31-865-s001.docx]

| **Acupoints** | **Depth of placement (mm / cun†)** | **Goal of therapy** |
| --- | --- | --- |
| Du20 | 0.3–0.5 inches | • Stimulate aspects of pain relief and healing • Calm spirit and uplift the patient’s mood • Sedation, easing mind, relieving pain, clearing heat |
| Li4 | 0.5–1 inches | • Move qi and blood opening the four gates • General pain reduction |
| Liv3 | 0.3–0.5 inches | • Move qi and blood opening the four gates • General pain reduction |
| Gb34 | 0.8–1.2 inches | • Enhance state of tendons and ligaments • Influential points for tendons • Improve weakness or numbness of lower extremities • Reduce pain and swelling in knee |
| Sp6 | 0.5–1.0 inches | Specific Ankle pain‡ |
| Li11 | 0.5–1.0 inches | Specific Elbow pain‡ |
| St35 | 0.5–1.2 inches | Specific Knee pain‡ |
| Kid3 | 0.3–0.5 inches | Specific Ankle pain‡ |
| Ear Shen Men | Stimulate the acupuncture point, but nottogo completely through the ear tissue | • Stimulate aspects of pain relief and healing • Calm spirit and uplift the patient’s mood • Sedation, easing mind, relieving pain, clearing heat |
| Xi yan | 0.5–1.0 inch bilaterally | Specific Knee pain |
| Heding | 0.3–0.5 inches | Specific Knee pain |
| Liv 8 | 0.5–0.8 inches | Specific Knee pain |
| Sp10 | 0.5–1.2 inches | Specific Knee pain |
| St 41 | 0.5–0.7 inches | Specific Ankle pain |
| Gb 40 | 0.5–0.8 inches | Specific Ankle pain |
| Sp5 | 0.2–0.3 inches | Specific Ankle pain‡ |
| Gb23 | 1.2 inches | Specific Lower back pain |
| Du4 | 0.5–1.0 inches | Specific Lower back pain‡ |
| Lu5 | 0.5–1.0 inches | Specific Elbow Pain |
| SJ5 | 0.5–10 inches | Specific Elbow Pain |

**Table III.** *Lambing et al.(13)*

† A cun often glossed as the Chinese inch, is**a traditional Chinese unit of length;**

‡ In instances where the studies did not provide specific details about placement depth or therapy goals, general informations have been presented based on MTC knowledge to ensure standardization.

| **Acupoints** | **Depth of placement (mm / cun†)** | **Goal of therapy** |
| --- | --- | --- |
| St 40 | Perpendicular/oblique insertion 1 to 1.5 cun‡ | *To unclog the energy channels* |
| St32 | Perpendicular or oblique insertion 1 to 2 cun‡ | *To unclog the energy channels* |
| St 41 | Perpendicular/oblique insertion 0.5 cun/ below the tendon‡ | *To unclog the energy channels* |
| Li 4 | Perpendicular/oblique insertion 0,5 to 1 cun/1 to 1.5 cun‡ | *To promote energy circulation* |
| Lu 9 | Perpendicular insertion 0.3 to 0.5 cun‡ | *To promote energy circulation* |
| Lr 3 | Oblique insertion 0.5 to 1.5 cun‡ | *To tonify the energy channels* |
| Sp 6 | Perpendicular/oblique insertion 1 to 1.5 cun‡ | *To tonify the energy channels* |
| Li 11 | Perpendicular insertion 1 to 1.5 cun‡ | *For energy flow harmonization* |
| Lr 8 | Perpendicular/oblique insertion 1 to 1.5 cun‡ | *For energy flow harmonization* |

**Table IV.** *Oliveira et al.(6)*

† A cun often glossed as the Chinese inch, is**a traditional Chinese unit of length;**

‡ In instances where the studies did not provide specific details about placement depth or therapy goals, general informations have been presented based on MTC knowledge to ensure standardization.

| **Acupoints** | **Depth of placement (mm / cun†)** | **Goal of therapy** |
| --- | --- | --- |
| Lu 5 | Perpendicular insertion 0.5 to 1 cun‡ | Specific Elbow Pain |
| Lu 11 | Perpendicular/oblique insertion 0.1 to 0.2 cun‡ | Specific Elbow Pain |
| Li10 | Perpendicular/oblique insertion 0.5 to 1.5 cun‡ | Specific Elbow Pain |
| Sp 9 | Perpendicular insertion 1 to 1.5 cun | Specific Elbow Pain |
| Sp 10 | Perpendicular/oblique insertion 1 to 1.5 cun‡ | Specific Elbow Pain |
| Lr 8 | Perpendicular/oblique insertion 1 to 1.5 cun‡ | Specific Elbow Pain |

**Table V.** *Rosted et al.(17)*

† A cun often glossed as the Chinese inch, is**a traditional Chinese unit of length;**

‡ In instances where the studies did not provide specific details about placement depth or therapy goals, general informations have been presented based on MTC knowledge to ensure standardization.

| **Acupoints** | **Depth of placement (mm / cun†)** | **Goal of therapy** |
| --- | --- | --- |
| Du 18 | Transverse insertion 0.5 to 1 cun‡ | General Management of Pain‡ |

**Table VI.** *Wallny et al.(18)*

† A cun often glossed as the Chinese inch, is**a traditional Chinese unit of length;**

‡ In instances where the studies did not provide specific details about placement depth or therapy goals, general informations have been presented based on MTC knowledge to ensure standardization.
